# Supplementary material for: Insulin/Snail1 axis ameliorates fatty liver disease by epigenetically suppressing lipogenesis
Source: Nat Commun. 2018 Jul 16;9:2751. doi: 10.1038/s41467-018-05309-y (PMC6048127; doi:10.1038/s41467-018-05309-y)
Supplement: Supplementary file 1 — Supplementary Information [file 41467_2018_5309_MOESM1_ESM.pdf]

Insulin/Snail1 Axis Ameliorates Fatty Liver Disease by Epigenetically Suppressing Lipogenesis

Liu Y *et. al.*

Supplementary Figures

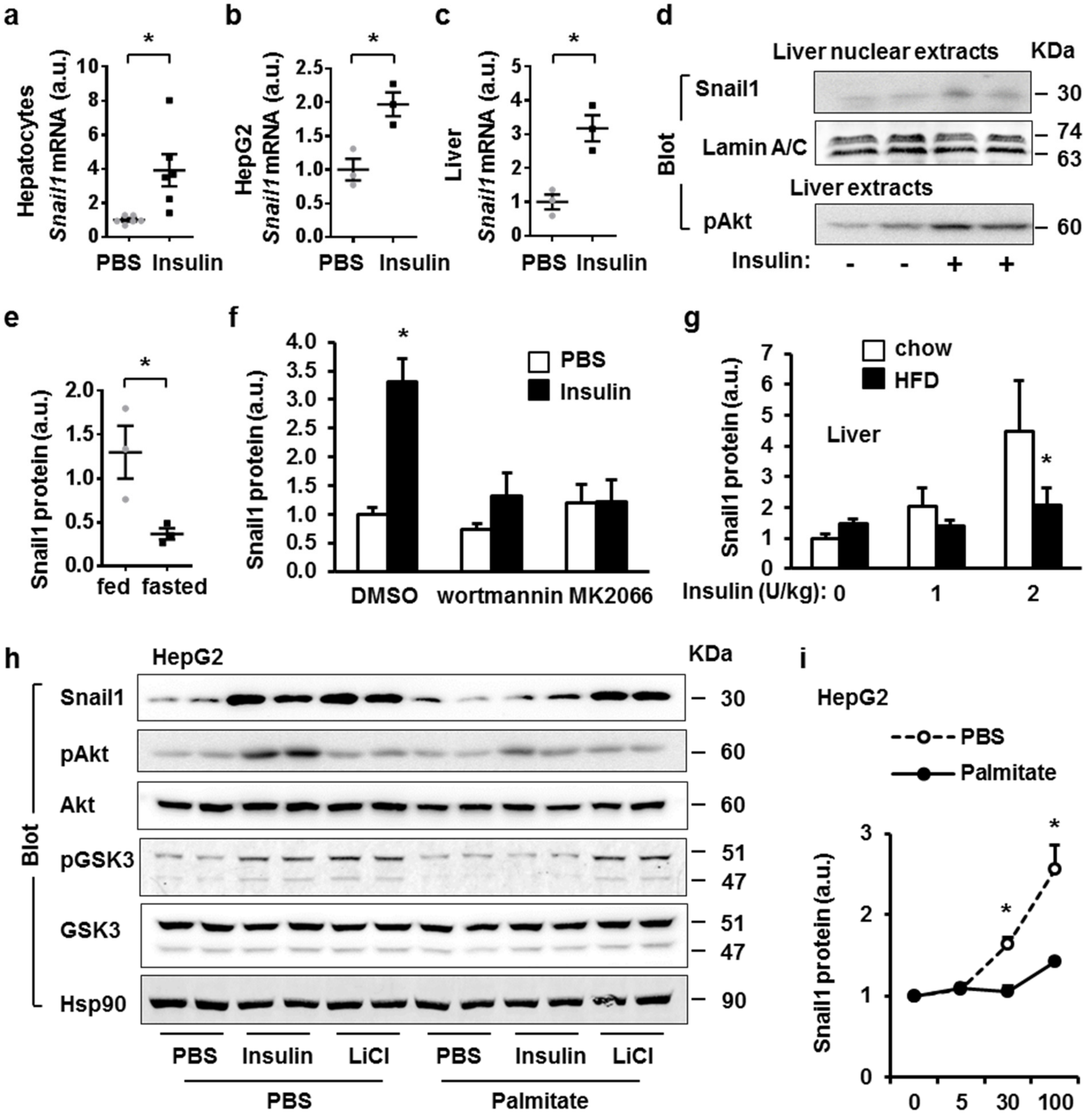

Supplementary Figure 1 Insulin upregulates hepatic Snail1. **a** Primary hepatocytes were deprived of serum overnight and stimulated with 50 nM insulin or PBS for 2 h. *Snail1* mRNA abundance was quantified by qPCR and normalized to 36B4 levels (n=4). **b** HepG2 cells were deprived of serum overnight and stimulated with 100 nM insulin for 2 h. *Snail1* mRNA levels were quantified by qPCR (normalized to GAPDH levels; n=3). **c-d** C57BL/6 male mice (8-10 wks) were fasted overnight and stimulated with insulin (0.5 units/kg body weight, i.p.) or PBS for 2 h. **c** Liver *Snail1* mRNA levels were quantified by qPCR (normalized to 36B4 levels; n=3). **d** Liver nuclear extracts were immunoblotted with antibodies against Snail1 and lamin A/C. **e** Mouse liver nuclear Snail1 levels (normalized to lamin A/C) at the fed and the overnight fasted states (n=5). **f** Snail1 protein in Fig. 1a was normalized to Hsp90 levels. **g** C57BL/6 mice (n=3) were fed a chow or HFD diet and treated with insulin at the indicated doses for 2 h. Liver nuclear Snail1 levels were normalized to lamin A/C levels. **h-i** HepG2 cells were pretreated with palmitate (100 µM) overnight. **h** The cells were then stimulated with insulin (100 nM) or LiCl (25 mM) for 2 h. Cell extracts were immunoblotted with the indicated antibodies. **i** The cells were then stimulated with insulin at the indicated concentrations for 2 h. Snail1 levers were quantified (normalized to Hsp90 levels) (n=3). Data are presented as mean ± SEM. *p<0.05, 2-tailed unpaired Student's *t* test.

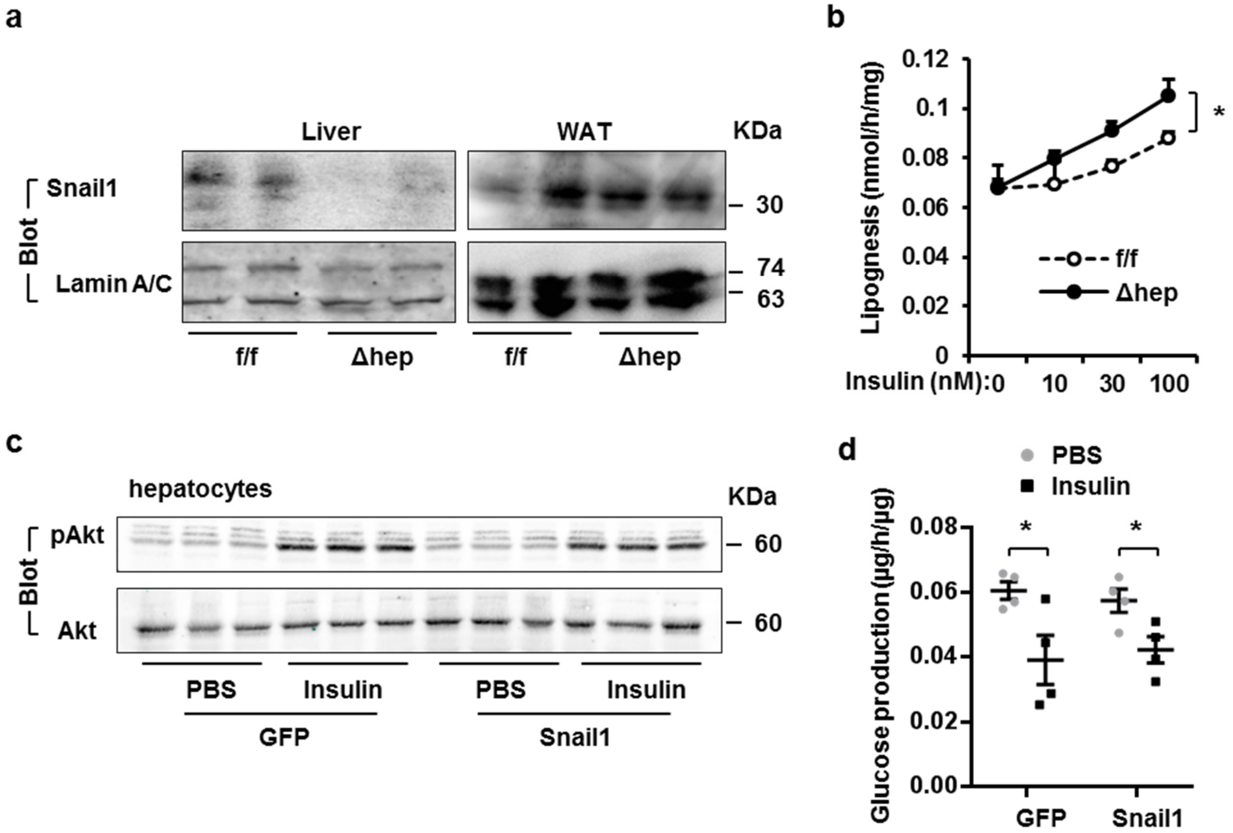

Supplementary Figure 2 Snail1 does not directly regulate insulin signaling and gluconeogenesis in hepatocytes. **a** *Snail1$^{flox/flox}$* (f/f) and *Snail1$^{flox/flox}$*;*CreERT2* (Δhep) males were treated with tamoxifen. Liver and WAT nuclear extracts were isolated 2 wks after tamoxifen treatment and immunoblotted with the indicated antibodies. **b** Primary hepatocytes were stimulated with insulin for 12 h, and lipogenesis were measured. Data were analyzed using ANOVA. **c-d** Primary hepatocytes were transduced with GFP or Snail1 adenoviral vectors. **c** The cells were deprived of serum overnight and stimulated with 100 nM insulin for 30 min. Cell extracts were immunoblotted with anti-phospho-Akt (pSer473) or anti-Akt antibodies. **d** The cells were deprived of serum overnight and stimulated with 100 nM insulin or PBS for 6 h in KRB buffer. Glucose production (n=4) was measured and normalized to total protein levels as we described previously (*Liu et al. Endocrinology,158:1207-1216, 2017*). Data are presented as mean ± SEM. *p<0.05, 2-tailed unpaired Student's *t* test or ANOVA.

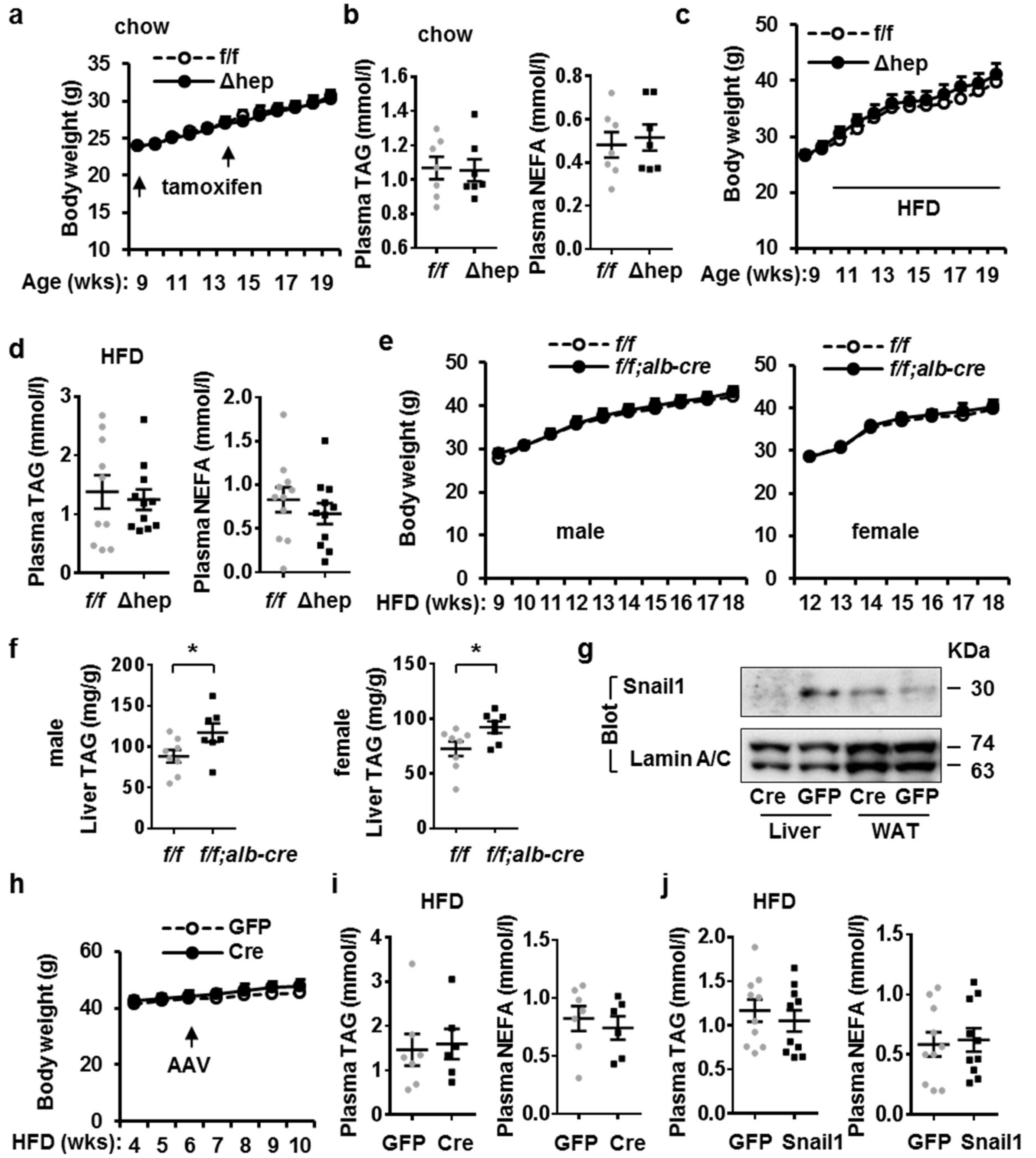

Supplementary Figure 3 Hepatocyte-specific deletion of *Snail1* promotes NAFLD. **a**-**b** *Snail1*$^{flox/flox}$ (f/f; n=7) and *Snail1*$^{flox/flox}$;*CreERT2* (Δhep; n=8) males were treated with tamoxifen twice as indicated, and growth curves were monitored. **b** Plasma TAG and NEFA levels. **c**-**d** *Snail1*$^{flox/flox}$ (n=11) and *Snail1*$^{\Delta hep}$ (n=11) male littermates were fed a HFD for 10 wks. **c** Growth curves. **d** Plasma TAG and NEFA levels. **e-f** *Snail1*$^{flox/flox}$ (male: n=8, female: n=8) and *Snail1*$^{flox/flox}$;*albumin-cre* (male: n=8, female: n=7) mice were fed a HFD for 10 wks. **e** Body weight. **f** Liver TAG levels. **g-i** *Snail1*$^{flox/flox}$ males were fed a HFD for 6 wks, transduced with AAV-GFP (n=7) or AAV-Cre (n=6) vectors, and fed a HFD for additional 4 wks. **g** Liver and WAT nuclear extracts were immunoblotted with the indicated antibodies. **h** Grown curves. AAV transduction time was marked. **i** Plasma TAG and NEFA levels. **j** C57BL/6 mice fed a HFD and transduced with GFP (n=9) or Snail1 (n=10) adenoviral vectors as described in Fig. 4a. Plasma TAG and NEFA levels were measured 3 wks after transduction. Data are presented as mean ± SEM.

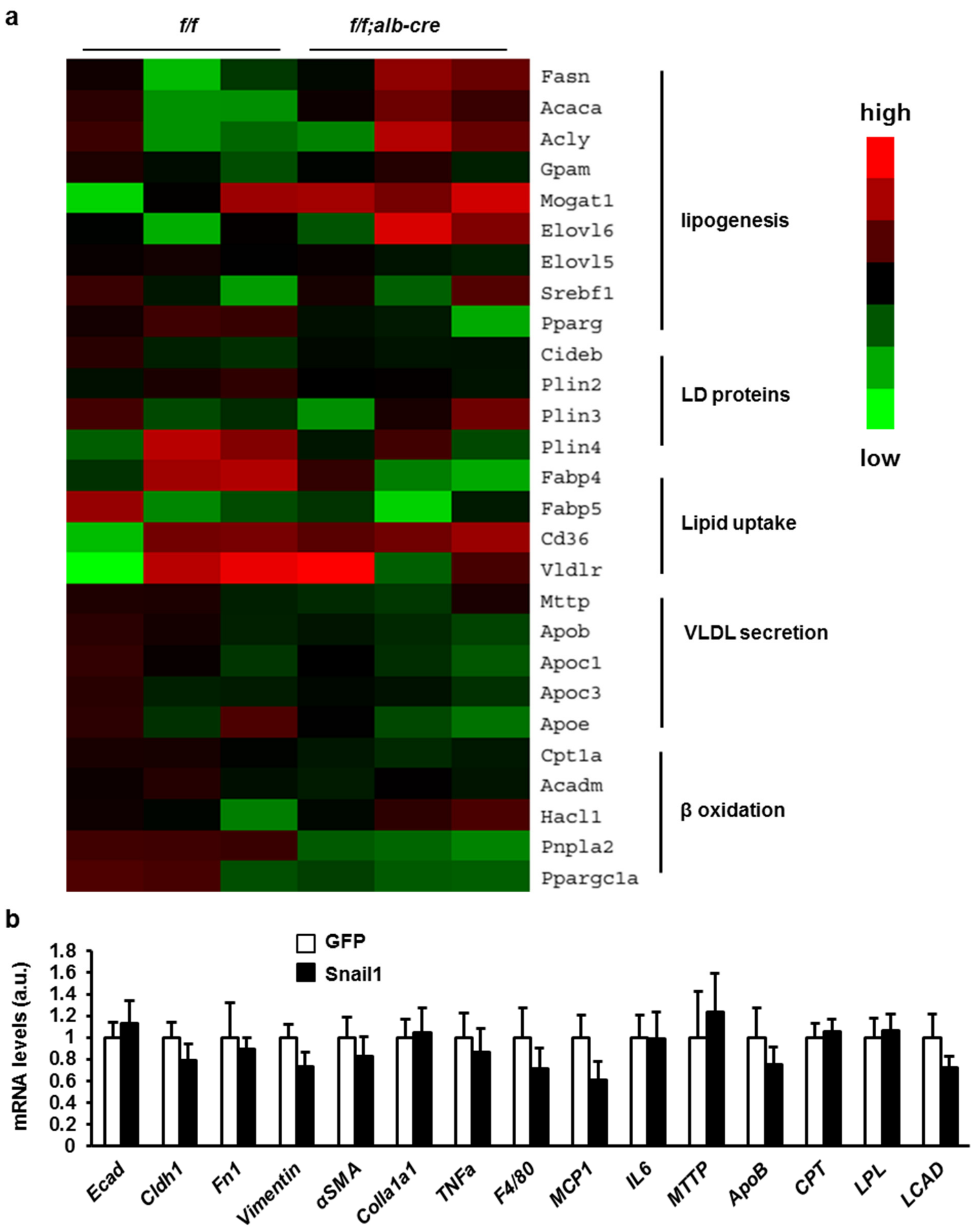

Supplementary Figure 4 Hepatocyte Snail1 suppresses the hepatic lipogenic program. **a** *Snail1*$^{flox/flox}$ and *Snail1*$^{flox/flox}$*;albumin-Cre* male mice were fed a HFD for 10 wks. Livers were harvested under non-fasting conditions and subjected to Affymetrix GeneChip Arrays analysis. Gene expression was presented using a heatmap. **b** C57BL/6 mice were fed a HFD for 6 wks and transduced with GFP (n=5) or Snail1 (n=5) adenoviral vectors. Liver gene expression was measured by qPCR 4 wks later, and normalized to 36B4 levels. Data are presented as mean ± SEM.

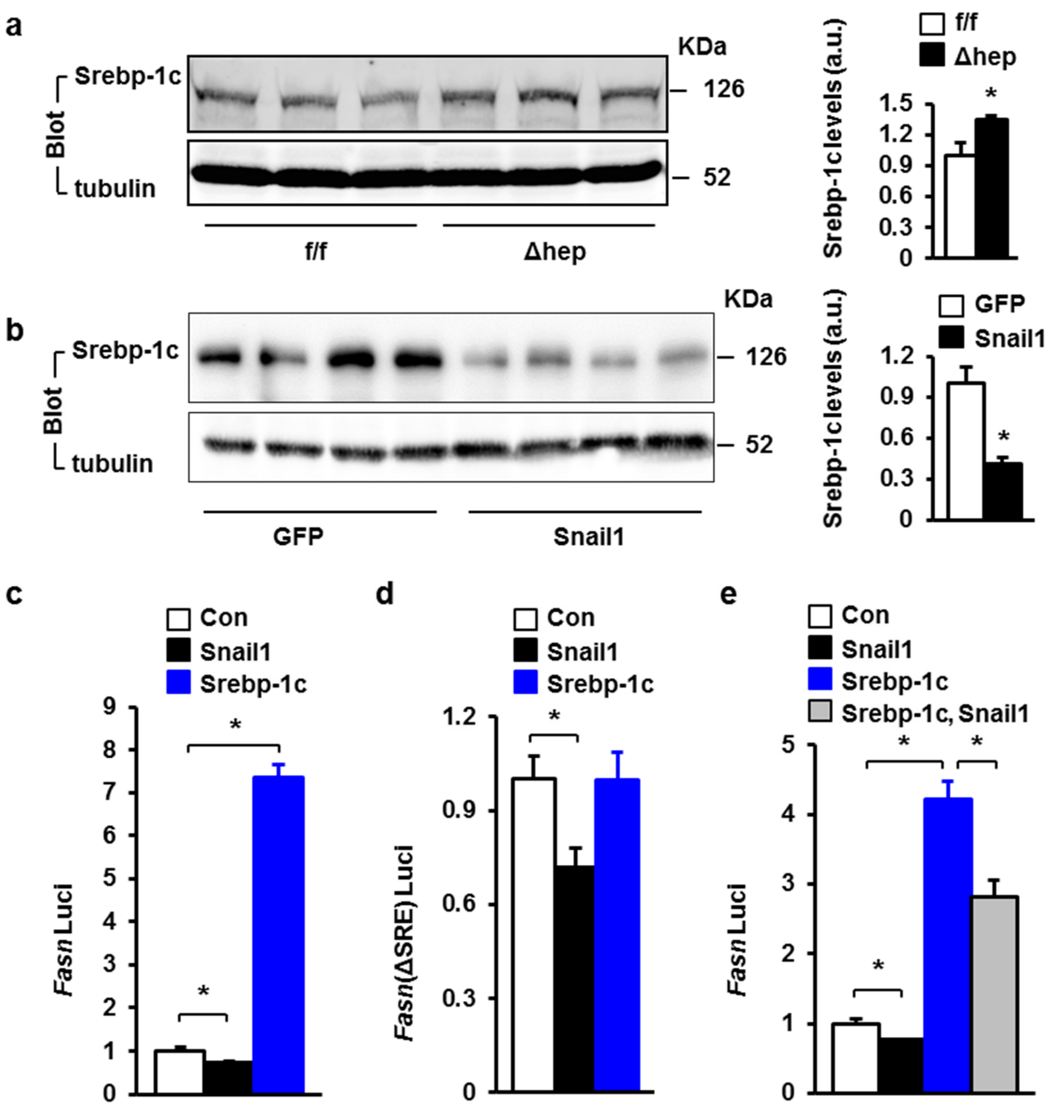

Supplementary Figure 5 Snail1 downregulates Srebp-1c in the liver. **a** *Snail1*$^{flox/flox}$ and *Snail1*$^{\Delta hep}$ male littermates were fed a HFD for 10 wks. Liver extracts were immunoblotted with antibodies against Srebp-1c and α-tubulin. Srebp-1c levels were normalized to α-tubulin levels. **b** C57BL/6 mice were fed a HFD for 7 wks and transduced with GFP or Snail1 adenoviral vectors for 3 wks. Liver Srebp-1c levels were normalized to α-tubulin levels. **c**-**e** *Fasn* and *Fasn* (ΔSRE) luciferase activities in HepG2 cells transfected with the indicated expression plasmids. Data are presented as mean ± SEM. *p<0.05, 2-tailed unpaired Student's *t* test.

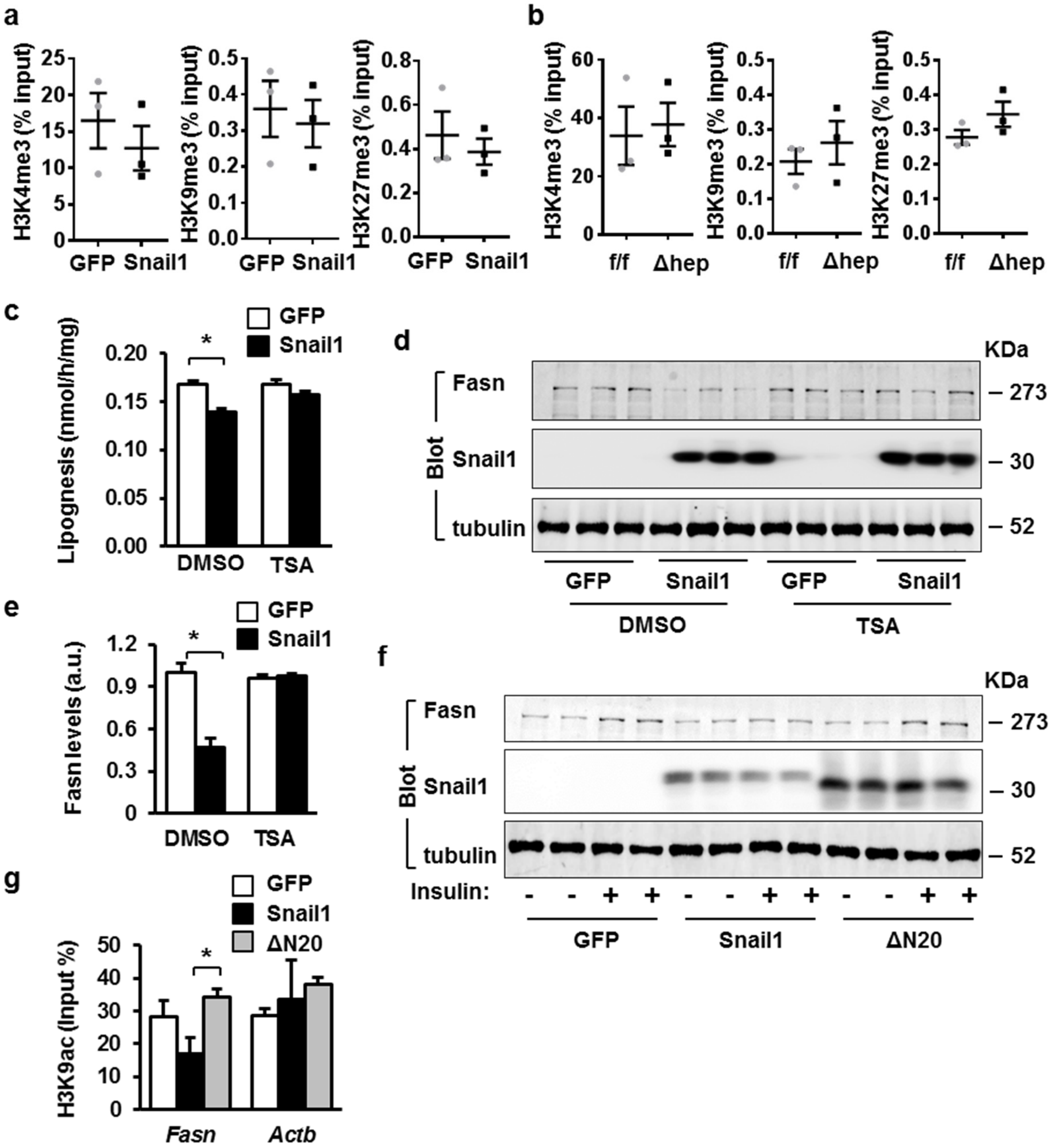

Supplementary Figure 6 Snail1 epigenetically suppresses hepatic *Fasn* expression. **a** C57BL/6 male mice (8-9 wks) were fed a HFD for 7 wks and then transduced with Snail1 (n=3) or GFP (n=3) adenoviral vectors. Livers were harvested 3 wks after transduction. The levels of H3Kme3, H3K9me3, and H3K27me3 on the *Fasn*, *NCH*, or *Actb* promoter were measured in the liver by ChIP-qPCR and normalized to inputs. **b** *Snail1*$^{\Delta hep}$ (n=3) and *Snail1*$^{flox/flox}$ (n=3) males were fed a HFD for 10 wks. The levels of H3K4me3, H3K9me3, and H3K27me3 on the *Fasn*, *NCH*, or *Actb* promoter were measured in the liver and normalized to inputs. **c**-**e** Primary hepatocytes were transduced with Snail1 and treated with TSA. **c** Lipogenesis (normalized to protein levels) (n=3). **d-e** Hepatocyte extracts were immunoblotted with the indicated antibodies. Fasn levels were normalized to α-tubulin levels. **f** Primary hepatocytes were transduced with Snail1 or ΔN20 adenoviral vectors for 24 h, and then stimulated with insulin (50 nM) for 12 h. Cell extracts were immunoblotted with the indicated antibodies. **g** Primary hepatocytes were transduced with Snail1, ΔN20, or GFP adenoviral vectors. H3K9ac levels on the *Fasn* promoter were measured by ChIP. Data are presented as mean ± SEM. *p<0.05, 2-tailed unpaired Student's *t* test.

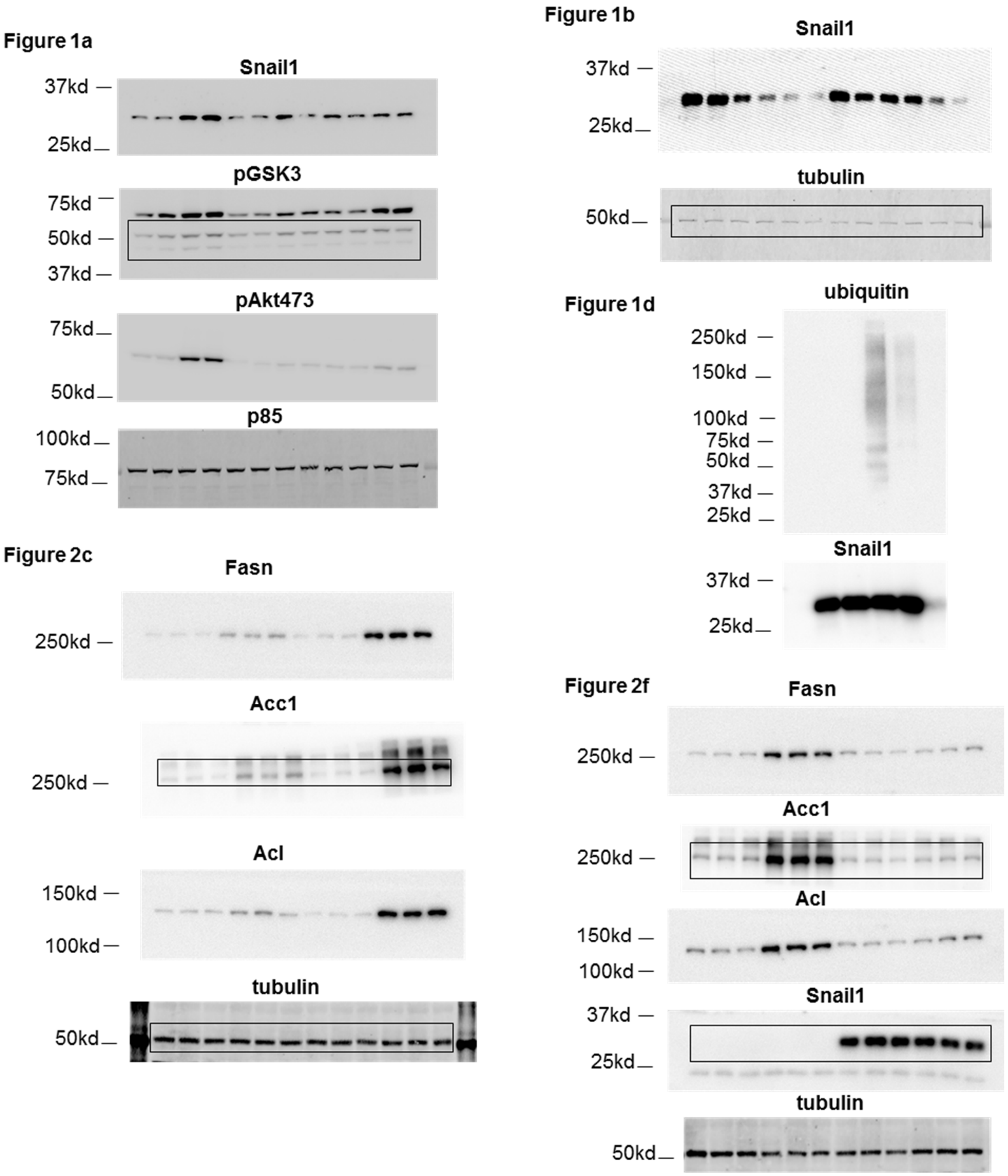

Supplementary Figure 7 The uncropped scans of western blots and gels for Figures 1-2.

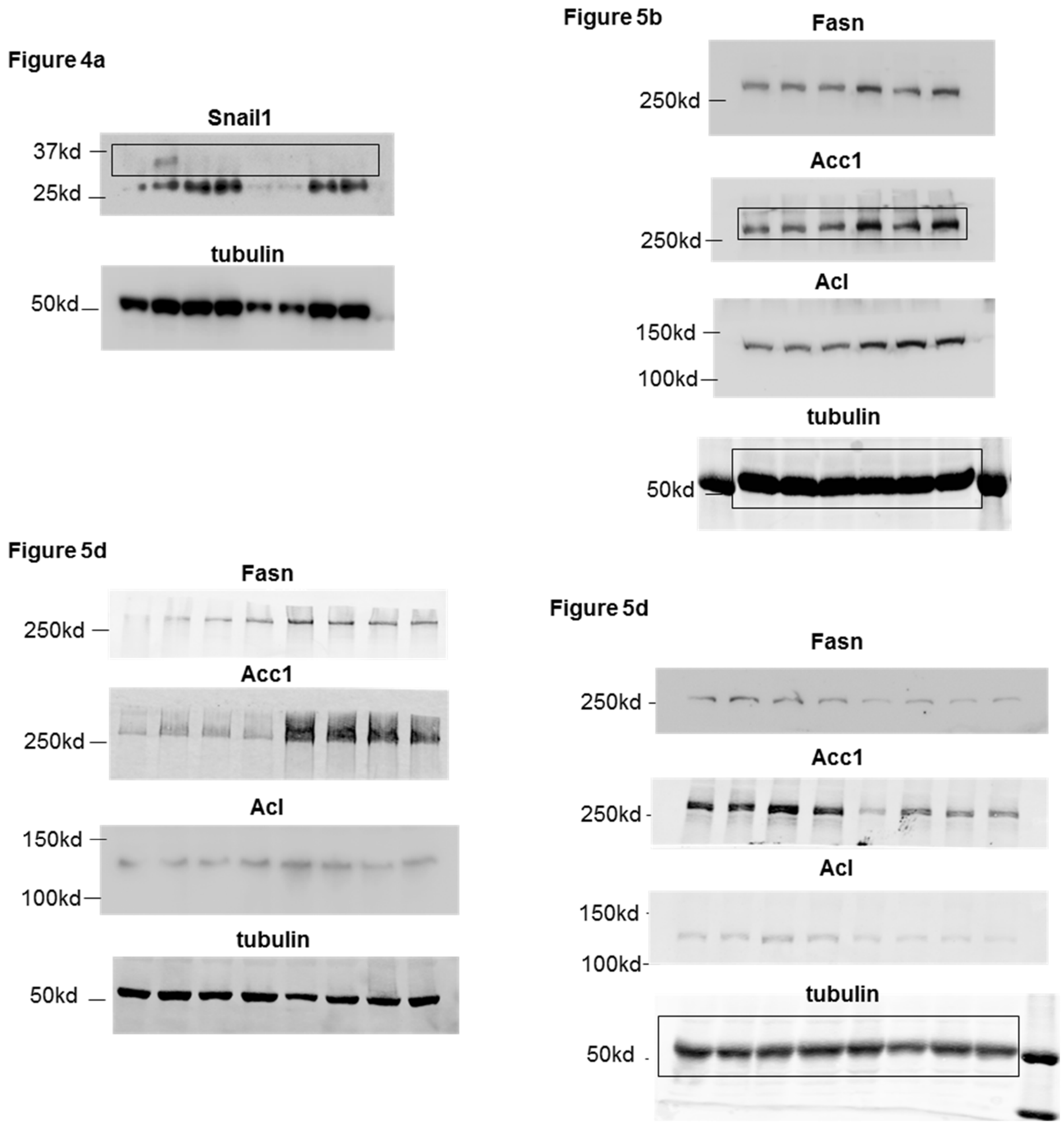

Supplementary Figure 8 The uncropped scans of western blots and gels for Figures 4-5.

Figure 6a

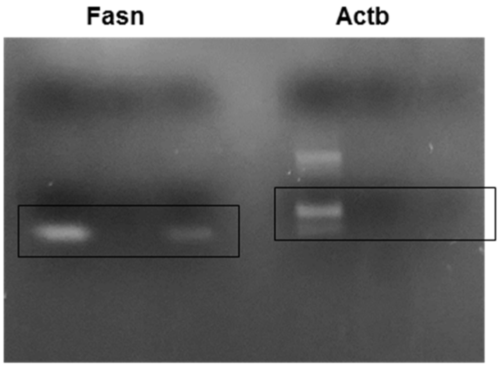

Figure 6c

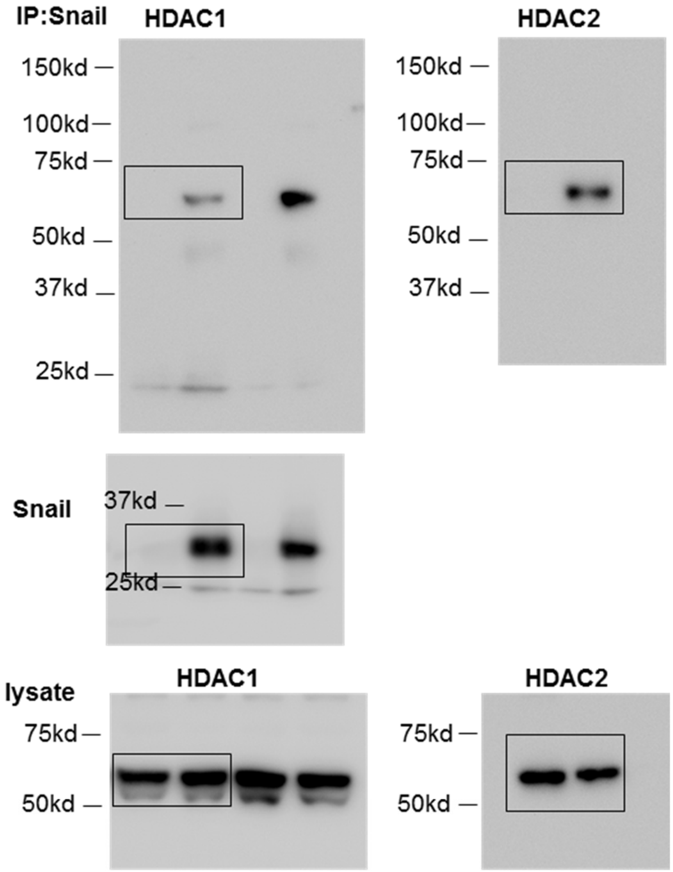

Figure 7d

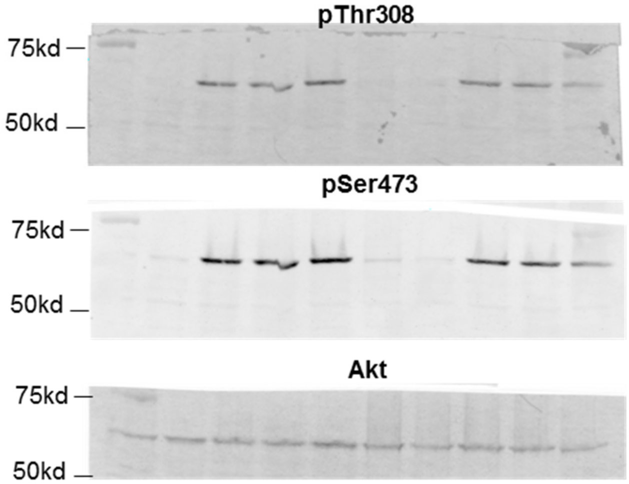

Supplementary Figure 9 The uncropped scans of western blots and gels for Figures 6-7.

Supplementary Table 1 Antibodies

ANTIBODY	SOURCE	IDENTIFIER	DILUTION
Snail1	Cell Signaling Technology	#3895	1:1000
Lamin A/C	Cell Signaling Technology	#4777	1:2000
p-Akt (pThr308)	Cell Signaling Technology	#13038	1:5000
p-Akt (pSer473)	Cell Signaling Technology	#4060	1:5000
Akt	Cell Signaling Technology	#4685	1:2000
α-tubulin	Santa Cruz	sc-5286	1:10000
Fasn	Cell Signaling Technology	#3180	1:5000
ACC1	Cell Signaling Technology	#3676	1:5000
ACL	Cell Signaling Technology	#13390	1:5000
HDAC1	Cell Signaling Technology	#5356	1:1000
HDAC2	Cell Signaling Technology	#5113	1:1000
Snail1	Cell Signaling Technology	#3879	1:200
H3K9ac	Cell Signaling Technology	#9649	1:200
H3K9me3	Cell Signaling Technology	#13969	1:200
H3K27ac	EMD Millipore	07-360	1:200
H3K27me3	Cell Signaling Technology	#9733	1:200
H3K4me3	Cell Signaling Technology	#9751	1:200
pGSK3	Cell Signaling Technology	#8566	1:3000
GSK3	Cell Signaling Technology	#5676	1:3000
HSP90	Cell Signaling Technology	#4877	1:5000
HA	U-M Hybridoma Core	12C5	1:1000
Ubiquitin	Santa Cruz	sc-8017	1:1000
Srebp-1c	Santa Cruz	Sc-13551	1:500

Supplementary Table 2 qPCR and ChIP primers

Genes	Forward	Reverse
Snail1 (mouse)	CCTTGTGTCTGCACGACCTGT	CACTGGTATCTCTTCACATCCG
Snail1 (human)	TCGGAAGCCTAACTACAGCGA	AGATGAGCATTGGCAGCGAG
Fasn	TTGACGGCTCACACACCTAC	CGATCTTCCAGGCTCTTCAG
ACC1	CAGGGACTATGTCCTGAAGCA	GGAATCCATTGTGGAGAGGA
Srebp-1c	AACGTCACTTCCAGCTAGAC	CCACTAAGGTGCCTACAGAGC
Lxrα	GAGTTCTCCAGAGCCATGAATG	ATATGTGTGTTGCAGCCTCTCT
MTTP	CTCCACAGTGCAGTTCTCACA	AGAGACATATCCCCTGCCTGT
apoB	CCAGAGTGTGGAGCTGAATGT	TTGCTTTTTAGGGAGCCTAGC
Cpt1α	CTGATGACGGCTATGGTGTTT	GTGAGGCCAAACAAGGTGATA
LPL	AGAAGGGAAAGGACTCAGCAG	TCAAACACCCAAACAAGGGTA
LCAD	CACTCAGATATTGTCATGCCCT	TCCATTGAGAATCCAATCACTC
Ecad	ACCGGAAGTGACTCGAAATG	GCTGCCTTCAGGTTTTCATC
Cldh1	GGGGACAACATCGTGACCG	AGGAGTCGAAGACTTTGCACT
Fn1	ATGTGGACCCCTCCTGATAGT	GCCCAGTGATTTCAGCAAAGG
vimentin	GACCTCACTGCTGCCCTGCG	GACTCCTGCTTGGCCTGGCG
αSMA	GTTCAGTGGTGCCTCTGTCA	ACTGGGACGACATGGAAAAG
Colla 1a1	TCACCTACAGCACCCTTGTG	GGTGGAGGGAGTTTACACGA
TNFα	CATCTTCTCAAAATTCGAGTGACAA	TGGGAGTAGACAAGGTACAACCC
F4/40	CTTTGGCTATGGGCTTCCAGTC	GCAAGGAGGACAGAGTTTATCGTG
MCP1	ACTGAAGCCAGCTCTCTCTTCCTC	TTCCTTCTTGGGGTCAGCACAGAC
IL6	AGCCAGAGTCCTTCAGA	GGTCCTTAGCCACTCCT
ACL	CCTCAAGGACTTCGTCAAACA	GCCCATACTCCTTCCTAGCAC
34B4	AAGCGCGTCCTGGCATTGTCT	CCGCAGGGGCAGCAGTGGT
GAPDH (human)	CGACCACTTTGTCAAGCTCA	AGGGGTCTACATGGCAACTG
Fasn (ChIP)	TGCAACCGTAGTCCAACGAG	GCCTCAGCGGAAGTCATCAG
NCH (ChIP)	GGATGGCTCCAAGATAAGGCA	ACAACATCCACACGTCCAGT
Actb (ChIP)	AATAGCCTCCGCCCTTGTG	CGTGACATCCACACCCAGA
