# Supplementary material for: Insulin/Snail1 axis ameliorates fatty liver disease by epigenetically suppressing lipogenesis
Source: Nat Commun. 2018 Jul 16;9:2751. doi: 10.1038/s41467-018-05309-y (PMC6048127; doi:10.1038/s41467-018-05309-y)
Supplement: Supplementary file 3 — Description of Additional Supplementary Files [file 41467_2018_5309_MOESM3_ESM.pdf]

Description of Additional Supplementary Files

File Name: Supplementary Data 1

Description: The raw data. Fig. 2, Fig. 5, and Supplementary Fig. 6e: The mRNA levels of Fasn, Acc1, and Acl were normalized to 36B4 levels (internal control). The values were presented as fold changes relative to control groups, respectively. Fig. 6f and Supplementary Fig. 6c: Lipogenesis rates were normalized to protein levels. Fig. 6g and Supplementary Fig. 6g, H3K27ac and H3K9ac levels on the Fasn promoter were presented as the percentage of input. Fig. 7: AUC fold changes (relative to control groups) in GTT and ITT. Supplementary Fig. 1: Snail1 protein levels were quantified by ImageJ and normalized to Hsp90 levels (f) or Lamin A/C (g). Fold changes were presented (relative to control groups). Supplementary Fig. 5: Fasn and Fasn (ΔSRE) luciferase activities were normalized to β-gal (internal control). Values are presented as fold changes relative to control groups.
